# Effects of Choice of Medical Imaging Modalities on a Non-invasive Diagnostic and Monitoring Computational Framework for Patients With Complex Valvular, Vascular, and Ventricular Diseases Who Undergo Transcatheter Aortic Valve Replacement

**DOI:** 10.3389/fbioe.2021.643453

**Published:** 2021-07-08

**Authors:** Melissa Baiocchi, Shirley Barsoum, Seyedvahid Khodaei, Jose M. de la Torre Hernandez, Sydney E. Valentino, Emily C. Dunford, Maureen J. MacDonald, Zahra Keshavarz-Motamed

**Affiliations:** ^1^Department of Mechanical Engineering, McMaster University, Hamilton, ON, Canada; ^2^Hospital Universitario Marques de Valdecilla, IDIVAL, Santander, Spain; ^3^Department of Kinesiology, McMaster University, Hamilton, ON, Canada; ^4^School of Biomedical Engineering, McMaster University, Hamilton, ON, Canada; ^5^School of Computational Science and Engineering, McMaster University, Hamilton, ON, Canada

**Keywords:** computational model, local hemodynamics, global hemodynamics, workload, diagnostic tool, doppler echocardiography, computed tomography

## Abstract

Due to the high individual differences in the anatomy and pathophysiology of patients, planning individualized treatment requires patient-specific diagnosis. Indeed, hemodynamic quantification can be immensely valuable for accurate diagnosis, however, we still lack precise diagnostic methods for numerous cardiovascular diseases including complex (and mixed) valvular, vascular, and ventricular interactions (C3VI) which is a complicated situation made even more challenging in the face of other cardiovascular pathologies. Transcatheter aortic valve replacement (TAVR) is a new less invasive intervention and is a growing alternative for patients with aortic stenosis. In a recent paper, we developed a non-invasive and Doppler-based diagnostic and monitoring computational mechanics framework for C3VI, called C3VI-DE that uses input parameters measured reliably using Doppler echocardiography. In the present work, we have developed another computational-mechanics framework for C3VI (called C3VI-CT). C3VI-CT uses the same lumped-parameter model core as C3VI-DE but its input parameters are measured using computed tomography and a sphygmomanometer. Both frameworks can quantify: (1) global hemodynamics (metrics of cardiac function); (2) local hemodynamics (metrics of circulatory function). We compared accuracy of the results obtained using C3VI-DE and C3VI-CT against catheterization data (gold standard) using a C3VI dataset (*N* = 49) for patients with C3VI who undergo TAVR in both pre and post-TAVR with a high variability. Because of the dataset variability and the broad range of diseases that it covers, it enables determining which framework can yield the most accurate results. In contrast with C3VI-CT, C3VI-DE tracks both the cardiac and vascular status and is in great agreement with cardiac catheter data.

## Introduction

Cardiovascular disease remains the primary cause of death worldwide and produces immense health and economic burdens (Roth et al., 2017; Ritchie and Roser, 2018; Benjamin et al., 2019). Cardiovascular disease is prevalent in 48.0% of adults and was responsible for 31.8% of all deaths in 2017 (Ritchie and Roser, 2018; Benjamin et al., 2019) and will remain the first cause of death globally by 2030. In the most general condition, several diseases of the valves, ventricles and the vascular system mechanically interact with one another and their combination exacerbate adverse effect of each isolated disease on the cardiovascular system (Généreux et al., 2013; Nombela-Franco et al., 2014; Blanke et al., 2016; Sotiropoulos et al., 2016). *This complex (and mixed) valvular, vascular and ventricular interactions (C3VI) is a complicated situation made even more challenging in the face of other cardiovascular pathologies.* C3VI represent situations in which a number of vascular, valvular and ventricular pathologies have mechanical interactions with each other. C3VI includes diseases of the heart valves such as stenosis and regurgitation of aortic and mitral valves, ventricular pathologies such as hypertrophy and heart failure, diseases of the vascular system such as hypertension as well as anatomical alterations due to interventions for C3VI such as transcatheter and surgical valve replacement (Elmariah et al., 2013; Généreux et al., 2013; Nombela-Franco et al., 2014; Pibarot et al., 2015; Ben-Assa et al., 2019).

*“Cardiology is flow”*(Richter and Edelman, 2006) and indeed quantifications of hemodynamics can be immensely valuable for precise diagnosis, however, we still lack precise diagnostic tools for various cardiovascular diseases (Di Carli et al., 2016). There has been an emerging conclusion by many researchers that valvular disease is a complex and mixed disease that also depends on the ventricle and the vascular system states (Yin, 1987; Burkhoff et al., 2005; Borlaug and Kass, 2008; Taylor and Steinman, 2010; Dweck et al., 2012; Antonini-Canterin et al., 2013; Keshavarz-Motamed et al., 2014, 2016; Ben-Assa et al., 2019; Ikonomidis et al., 2019; Keshavarz-Motamed et al., 2020; Sadeghi et al., 2020; Khodaei et al., 2021a, b). Indeed, the quantitative investigations of hemodynamics in patients with C3VI should take into account the interactive coupling of the valves, ventricle, and the vascular system. The conclusions and recommendations made in the previous studies can be boiled down to define the following two hemodynamics quantification capabilities that computational diagnostic frameworks are required to have to be clinically useful for patients with C3VI. The required quantities are local and global hemodynamics metrics (Yin, 1987; Burkhoff et al., 2005; Borlaug and Kass, 2008; Taylor and Steinman, 2010; Dweck et al., 2012; Antonini-Canterin et al., 2013; Ky et al., 2013; Keshavarz-Motamed et al., 2014, 2016; Ben-Assa et al., 2019; Ikonomidis et al., 2019; Seemann et al., 2019; Keshavarz-Motamed et al., 2020; Sadeghi et al., 2020) as follows: (1) *Metrics of circulatory function (local)*, e.g., fluid dynamics of the circulatory system, and (2) *Metrics of cardiac function (global)*, e.g., heart workload and its breakdown to the contributing disease components. Assessments of hemodynamics, if available, would offer valued information about the cardiac health condition and could be used for planning C3VI interventions and making critical clinical decisions with life-threatening risks. Presently, *there are no tools available to invasively or non-invasively quantify local and global hemodynamics.* Phase-contrast magnetic resonance imaging (MRI) can offer the fluid dynamics. However, MRI has a lower temporal resolution than doppler echocardiography (DE) (Elkins and Alley, 2007; Kilner et al., 2007). It is important to note that, due to the high risk of the magnetic field of the machine for patients with implanted devices, MRI cannot be used for patients with most implanted medical devices except safely for MRI-conditional devices (Orwat et al., 2014). Computed tomography (CT) has a high spatial resolution and can provide anatomical information with a high accuracy (Villarraga-Gómez et al., 2018), however, it has a low temporal resolution (Maleki and Esmaeilzadeh, 2012; Watson et al., 2018; Rehman and Makaryus, 2019) and cannot measure any (local and global) hemodynamic parameters. Furthermore, CT uses ionizing radiation (Burgstahler and Schroeder, 2007; Fleischmann et al., 2008) so receiving multiple scans increases the risk of developing cancer (Edwards and Arthurs, 2011; Pearce et al., 2012; Power et al., 2016; Rigsby et al., 2018). Cardiac catheterization is the gold standard for evaluating cardiac function but it is invasive and carries high risk (Omran et al., 2003) so it not practical for diagnosis in patients with cardiovascular diseases in regular clinical practice. Most importantly, cardiac catheterization offers access to flow and pressure only in very limited regions. Doppler echocardiography (DE) is risk-free, has high temporal resolution and can be used to investigate cardiac function in real time. Despite DE’s potential advantages, there is no DE methods to quantify global hemodynamics and there is no method to quantify local hemodynamics accurately.

In this work, we seek for a method that can quantify global hemodynamics in addition to measures of local hemodynamics. Currently only lumped-parameter models have these capabilities due to the complexity of the cardiovascular system and the unmanageable computational cost that 3-D models of hemodynamics in the entire cardiovascular system has. A diagnostic lumped parameter model framework that can quantify both *local* and *global* hemodynamics in patients with cardiovascular diseases should meet the following 2 conditions:

(1) The computational diagnostic framework should be developed based on the clinical patient-specific input parameters (e.g., hemodynamic metrics, clinical data and imaging). Upon development of a diagnostic lumped parameter model, its results should be validated against clinical data obtained using DE, MRI, and more specifically cardiac catheterization.
(2) The patient-specific input parameters for such development should be obtained *non-invasively* in each patient. It is critical to note that obtaining the input parameters invasively in patient refutes the entire purpose of the diagnostic computational mechanics framework.

There have been attempts for quantifying hemodynamics and for fundamental understanding of cardiovascular mechanics using lumped parameter modeling (Segers et al., 2003; Geven et al., 2004; Tanné et al., 2008; Garcia et al., 2009, 2005; Keshavarz-Motamed et al., 2011, 2014, 2015, 2016; Mynard et al., 2012; Broomé et al., 2013; de Canete et al., 2013; Revie et al., 2013; Benevento et al., 2015; Frolov et al., 2016; Mihalef et al., 2017; Duanmu et al., 2018; Li et al., 2018; Pant et al., 2018; Ben-Assa et al., 2019; Mao et al., 2019; Keshavarz-Motamed, 2020; Keshavarz-Motamed et al., 2020). All of these models [except (Keshavarz-Motamed et al., 2016; Keshavarz-Motamed, 2020)] cannot satisfy Requirements #1 and #2 above, although they were very important to provide fundamental understandings using idealized or hypothetical cases (Segers et al., 2003; Geven et al., 2004; Tanné et al., 2008; Garcia et al., 2009, 2005; Keshavarz-Motamed et al., 2011, 2014, 2015; Mynard et al., 2012; Broomé et al., 2013; de Canete et al., 2013; Revie et al., 2013; Benevento et al., 2015; Frolov et al., 2016; Mihalef et al., 2017; Duanmu et al., 2018; Li et al., 2018; Pant et al., 2018; Ben-Assa et al., 2019; Mao et al., 2019; Keshavarz-Motamed et al., 2020). Among all of the previous studies, the only lumped-parameter models that satisfy both Requirements #1 and #2 are one on coarctation of the aorta (Keshavarz-Motamed et al., 2016) and the other one on C3VI (Keshavarz-Motamed, 2020).

Transcatheter aortic valve replacement (TAVR) is an emerging minimally invasive intervention for patients with aortic stenosis across a broad risk spectrum. In this study, we contributed to proceeding computational mechanics as an influential revenue to augment clinical data and measurements, and medical imaging to develop diagnostic methods for monitoring, treatment planning and risk assessment in patients with C3VI who undergo TAVR in both pre and post TAVR states at no risk to the patient. *In patients with C3VI and TAVR, DE and CT are commonly used but MRI is not usually used due the risk of the magnetic field interactions with the implanted devices in the body of these patients.* We recently developed (Keshavarz-Motamed, 2020) a highly innovative, Doppler-based, non-invasive, image-based, patient-specific diagnostic and monitoring lumped parameter model framework for C3VI (called C3VI-DE) which uses input parameters, measured reliably using DE and a sphygmomanometer and satisfy both Requirements (#1 & #2). C3VI-DE, which has a lumped-parameter model at its core, quantifies (1) local hemodynamics (e.g., details of the physiological pulsatile flow and pressure in the heart and circulatory system); (2) global hemodynamics (e.g., cardiac function hemodynamic metrics, LV workload, instantaneous LV pressure and volume) and most importantly the individual share of each disease constituent on the global hemodynamics. *Currently, in clinical practice, none of these metrics can be acquired in patients non-invasively and if invasive procedures using cardiac catherization are conducted, the measured metrics are not complete.* Additionally, in the present work, we have developed another computational-mechanics framework for C3VI (called C3VI-CT). C3VI-CT uses the same lumped-parameter model core as C3VI-DE and was coupled with input parameters measured using CT and a sphygmomanometer (for simplicity, we called this latter framework C3VI-CT). The two frameworks differ in terms of the modality used for collecting the input parameters for the core lumped-parameter model. In the present work, we compared accuracy of the results obtained from C3VI-DE and C3VI-CT against cardiac catheterization data in forty-nine C3VI patients who underwent TAVR to determine which framework can yield the most accurate results. To the best of our knowledge, this is the first study that investigates the effects of choice of medical imaging modalities on the accuracy of a computational diagnostic framework for patients with C3VI in terms of local and global hemodynamic.

## Materials and Methods

**FIGURE 1:**
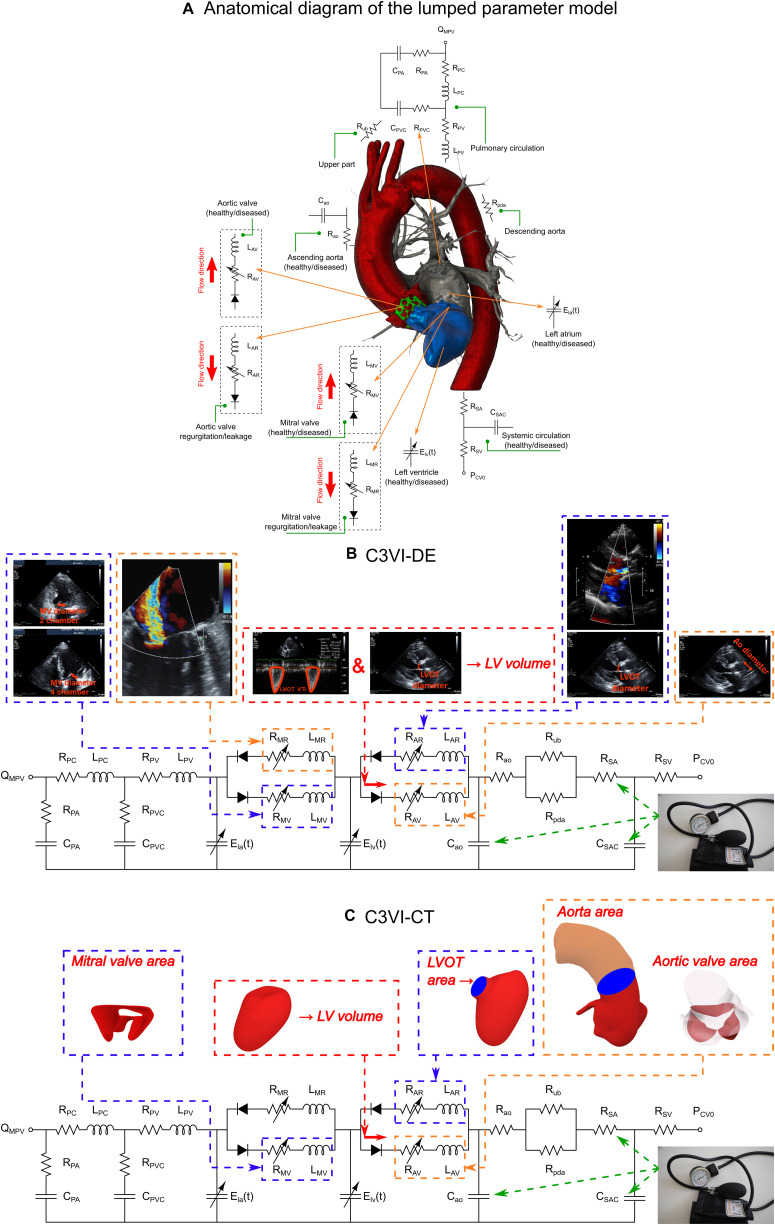
Schematic diagram of the lumped parameter modeling. **(A)** Anatomical representation. **(B)** Electrical representation of C3VI-DE. This model includes four sub-models. (1) left atrium, (2) left ventricle, (3) aortic valve, (4) mitral valve, (5) systemic circulation, and (6) pulmonary circulation (Table 1, abbreviations). C3VI-DE input parameters were measured using DE and sphygmomanometer. **(C)** Electrical representation of C3VI-CT. Input parameters of C3VI-CT were measured using CT and sphygmomanometer.

**FIGURE 2:**
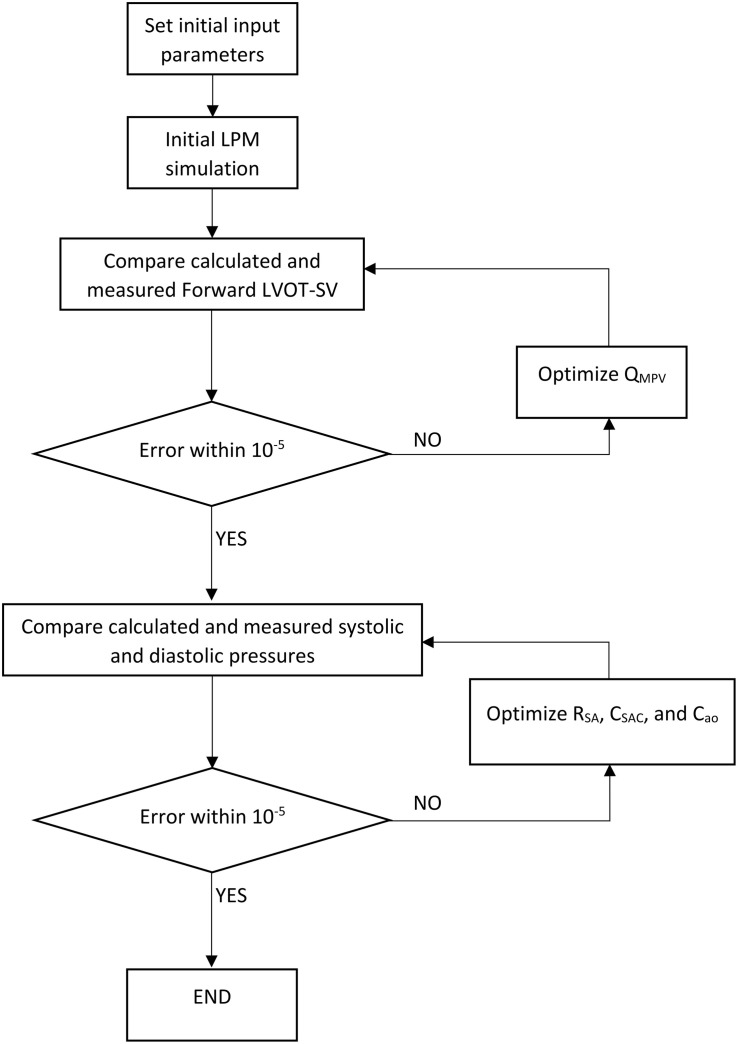
Patient-specific response optimization flow chart. This flow chart was used for both C3VI-DE and C3VI-CT.

Our recent non-invasive diagnostic and monitoring computational-mechanics framework for C3VI (called C3VI-DE) (Keshavarz-Motamed, 2020) uses limited input parameters, measured reliably using Doppler echocardiography and a sphygmomanometer. In this study, we have developed another computational-mechanics framework for C3VI (called C3VI-CT). C3VI-CT uses the same lumped-parameter model core as C3VI-DE and was coupled with input parameters measured using CT and a sphygmomanometer (Figure 1; Schematic diagrams of C3VI-DE and C3VI-CT). The developed algorithm (for both C3VI-DE and C3VI-CT) uses the following input parameters: systolic and diastolic brachial blood pressures, forward left ventricular outflow tract stroke volume, cardiac cycle duration, ejection time, ascending aorta area, left ventricular outflow tract area, aortic valve effective orifice area, mitral valve effective orifice area, and grading of the severity of aortic and mitral valves regurgitation. The algorithm consists of a parameter estimation algorithm and a lumped-parameter model that incorporates several sub-models to analyze any combination of mixed and complex valvular, vascular and ventricular diseases in both pre and post interventional status (see Figure 1 for Schematic diagrams; Figure 2 for Flow chart; Table 1 for Cardiovascular parameters).

**TABLE 1:** Cardiovascular parameters.

**Description**	**Abbreviation**	**Value**
**Valve parameters**		
Effective orifice area	EOA	Measured using DE and CT
Inertance (mitral valve)	M_*MV*_	Constant value: 0.53 gcm^–2^ (Flachskampf et al., 1993; Tanné et al., 2008; Keshavarz-Motamed, 2020) Defined by Flachskampf et al. (1993)
**Systematic circulation parameters**		
Aortic resistance	R_*ao*_	Constant value: 0.05 mmHg.s.mL^–1^ (Keshavarz-Motamed et al., 2011, 2014, 2016; Keshavarz-Motamed, 2020)
Aortic compliance	C_*ao*_	0.6 C_*SAC*_ (Stergiopulos et al., 1999) Initial value: 0.5 mL/mmHg (Keshavarz-Motamed et al., 2011, 2014, 2016; Keshavarz-Motamed, 2020) Optimized based on brachial pressures *(Systolic and diastolic brachial pressures are optimization constraints)*
Systemic vein resistance	R_*SV*_	0.05 mmHg.s.mL^–1^ (Keshavarz-Motamed et al., 2011, 2014, 2016; Keshavarz-Motamed, 2020)
Systemic arteries and veins compliance	C_*SAC*_	Initial value: 2 mL/mmHg (Keshavarz-Motamed et al., 2011, 2014, 2016; Keshavarz-Motamed, 2020) Optimized based on brachial pressures *(Systolic and diastolic brachial pressures are optimization constraints)*
systemic arteries resistance (including arteries, arterioles and capillaries)	R_*SA*_	Initial value: 0.8 mmHg.s.mL^–1^ (Keshavarz-Motamed et al., 2011, 2014, 2016; Keshavarz-Motamed, 2020) Optimized based on brachial pressures *(Systolic and diastolic brachial pressures are optimization constraints)*
Upper body resistance	R_*ub*_	Adjusted to have 15% of total flow rate in healthy case (Keshavarz-Motamed et al., 2016)
Proximal descending aorta resistance	R_*pda*_	Constant value: 0.05 mmHg.s.mL^–1^ (Keshavarz-Motamed et al., 2016; Keshavarz-Motamed, 2020)
Elastance Function*		
Maximum Elastance	E_*max*_	2.1 (LV) 0.17 (LA)
Minimum Elastance	E_*min*_	0.06 (LV, LA)
Elastance ascending gradient	m_1_	1.32 (LV, LA)
Elastance descending gradient	m_2_	27.4 (LV) 13.1 (LA)
Elastance ascending time translation	*τ*_1_	0.269 T (LV) 0.110 T (LA)
Elastance descending time translation	*τ*_2_	0.452 T (LV) 0.18 T (LA)
**Pulmonary circulation parameters**		
Pulmonary Vein Inertance	L_*PV*_	Constant value:0.0005 mmHg⋅s^2^⋅mL^–1^ (Tanné et al., 2008; Keshavarz-Motamed, 2020)
Pulmonary Vein Resistance	R_*PV*_	Constant value: 0.002 mmHg⋅s⋅mL^–1^ (Tanné et al., 2008; Keshavarz-Motamed, 2020)
Pulmonary Vein and capillary Resistance	R_*PVC*_	Constant value: 0.001 mmHg⋅s⋅mL^–1^ (Tanné et al., 2008; Keshavarz-Motamed, 2020)
Pulmonary Vein and Capillary Compliance	C_*PVC*_	Constant value: 40 mL/mmHg (Tanné et al., 2008; Keshavarz-Motamed, 2020)
Pulmonary Capillary Inertance	L_*PC*_	Constant value: 0.0003 mmHg⋅s^2^⋅mL^–1^ (Tanné et al., 2008; Keshavarz-Motamed, 2020)
Pulmonary Capillary Resistance	R_*PC*_	Constant value: 0.21 mmHg⋅s⋅mL^–1^ (Tanné et al., 2008; Keshavarz-Motamed, 2020)
Pulmonary Arterial Resistance	R_*PA*_	Constant value: 0.01 mmHg⋅s⋅mL^–1^ (Tanné et al., 2008; Keshavarz-Motamed, 2020)
Pulmonary Arterial Compliance	C_*PA*_	Constant value: 4 mL/mmHg (Tanné et al., 2008; Keshavarz-Motamed, 2020)
Mean Flow Rate of Pulmonary Valve	Q_*MPV*_	*Forward LVOT-SV* is the only input flow condition. *Q_*MPV*_ is a flow parameter that was optimized so that the lump-parameter model could reproduce the desirable measured Forward LVOT-SV*
**Input flow condition**		
Forward left ventricular outflow tract stroke volume	Forward LVOT-SV	Measured using DE and CT
**Output condition**		
Central venous pressure	P_*CV0*_	Constant value: 4 mmHg (Keshavarz-Motamed et al., 2011, 2014, 2016; Keshavarz-Motamed, 2020)
**Other**		
Constant blood density	ρ	Constant value: 1050 kg/m^3^ (Keshavarz-Motamed et al., 2011, 2014, 2016; Keshavarz-Motamed, 2020)
Cardiac cycle duration	T	Measured using DE and CT
Systolic End Ejection time	T_*EJ*_	Measured using DE and CT

*Summarized parameters used in the lumped parameter modeling to simulate all patient-specific cases.*

### Lumped Parameter Model

#### Cardiac-Arterial Model

##### Left ventricle

LV pressure and LV volume were coupled using a time varying elastance E(t) as follows:

(1) $$E\left(t\right)=\frac{P_{LV}\left(t\right)}{V\left(t\right)-V_{0}}$$

where, *P*_*LV*_(*t*), *V*(*t*), and *V*_0_ are the LV time-varying pressure, time-varying volume, and unloaded volume, respectively (Keshavarz-Motamed et al., 2016). As explained by Keshavarz-Motamed (2020), to represent the normalized elastance function of the LV, we observed that among summation of Gaussian functions (Pironet et al., 2013; Chaudhry, 2015), Boltzmann Distribution (McDowell, 1999), double Hill function (Mynard et al., 2012; Broomé et al., 2013), and the latter provided the most physiologically accurate results (e.g., pressure, volume, and flow waveforms). The double Hill function which is a cooperative process (Moss et al., 2004), as physiologically expected from myocyte recruitment during preload and is modeled by a sigmoidal Hill function.

(2) $$E\left(t\right)=N\left(\frac{{\left(\frac{t}{\tau_{1}}\right)}^{m_{1}}}{1+{\left(\frac{t}{\tau_{1}}\right)}^{m_{1}}}\right)\left(\frac{1}{1+{\left(\frac{t}{\tau_{2}}\right)}^{m_{2}}}\right)+E_{min}$$

(3) $$N=\frac{E_{max}-E_{min}}{2}$$

where, *N*, τ_1_, τ_2_, *m*_1_, *m*_2_, and *E*_*min*_ are elastane normalization, ascending time translation, descending time translation, ascending gradient, descending gradient, and minimum elastance, respectively (see Table 1). A double Hill function was modeled the contraction and relaxation in the heart chambers (equation 2); the first term in brackets resembles to the contraction of the chamber and the second term in brackets resembles to the relaxation of the chamber. τ_1_, τ_2_, *m*_1_, *m*_2_ govern the time translation and gradient of the elastance function, respectively: (1) τ_1_ and τ_2_ are parameters that are functions of the cardiac cycle duration (T) and are calculated in each patient using the equations provided in Table 1; (2) *m*_1_, *m*_2_ are constant for all patients (see Table 1 for more details). Parameter values used for the elastance function were adapted from Gleason and Braunwald (1962); Van de Werf et al. (1984); Brown and Ditchey (1988); Dell’Italia and Walsh (1988); Kass et al. (1988); Takeuchi et al. (1992); Senzaki et al. (1996); Stergiopulos et al. (1996); Maniar et al. (2003); Liang et al. (2009) to obtain physiologically waveforms (Table 1).

##### Left atrium

Left atrium pressure and LA volume were coupled using time varying elastance E(t), following the same method described above for the LV model (defined in equations 2 and 3) (Mynard et al., 2012; Broomé et al., 2013; Table 1). Additionally, a phase lag was used in the LA elastance function to account for the relative onset of contractions between LA and LV (Mynard et al., 2012). In Particular, LV contraction was introduced at T = 0, and LA contraction was launched at 0.85 T (Mynard et al., 2012), causing in a time delay of 0.15 T.

#### Modeling Heart Valves

##### Aortic valve

Aortic valve was modeled using the net pressure gradient formulation (*PG*_*net*_) through the aortic valve as follows:

(4) $${PG_{net}|}_{AV}=\frac{2\pi \rho }{\sqrt{{E_{L}Co|}_{AV}}}\frac{\partial Q\left(t\right)}{\partial t}+\frac{\rho }{{2E_{L}Co|}_{AV}^{2}}Q^{2}\left(t\right)$$

and

(5) $${E_{L}Co|}_{AV}=\frac{\left({EOA|}_{AV}\right)A_{AO}}{A_{AO}-{EOA|}_{AV}}$$

where, *E_L_Co*|_*AV*_, *EOA*|_*AV*_, *A*_*AO*_, ρ, and *Q*are the valvular energy loss coefficient, effective orifice area, ascending aorta cross sectional area, fluid density, and transvalvular flow rate, respectively.

##### Aortic regurgitation

Aortic regurgitation (AR) was modeled using the similar formulation as the aortic valve:

(6A) $${PG_{net}|}_{AR}=\frac{2\pi \rho }{\sqrt{{E_{L}Co|}_{AR}}}\frac{\partial Q\left(t\right)}{\partial t}+\frac{\rho }{{2E_{L}Co|}_{AR}^{2}}Q^{2}\left(t\right)$$

and

(6B) $${E_{L}Co|}_{AR}=\frac{EOA_{AR}A_{LVOT}}{A_{LVOT}-EOA_{AR}}$$

where, *E*_*L*_*Co*|_*AR*_, *EOA*_*AR*_, and *A*_*LVOT*_ are regurgitation energy loss coefficient, regurgitant effective orifice area and LVOT area, respectively. AR pressure gradient is the difference between aorta pressure and LV pressure during diastole.

##### Mitral valve

Mitral valve (MV) was modeled using the analytical formulation for the net pressure gradient (*PG*_*net*_|_*MV*_) across the MV during LA ejection. *PG*_*net*_|_*MV*_ was expressed as a function of ρ, *Q*_*MV*_, *EOA*_*MV*_ and *M*_*MV*_, represent the fluid density, transvalvular flow rate, effective orifice area and inertance, respectively.

(7) $${PG_{net}|}_{MR}=\frac{M_{MV}}{EOA_{MV}}\frac{\partial Q_{MV}\left(t\right)}{\partial t}+\frac{\rho }{{2EOA|}_{MV}^{2}}Q_{MV}^{2}\left(t\right)$$

##### Mitral regurgitation

Mitral regurgitation (MR) pressure gradient is the difference between mitral pressure and LA pressure during systole and was modeled using the following equation:

(8) $${PG_{net}|}_{MR}=\frac{M_{MV}}{EOA_{MR}}\frac{\partial Q\left(t\right)}{\partial t}+\frac{\rho }{{2EOA|}_{MR}^{2}}Q^{2}\left(t\right)$$

where, *EOA*|_*MR*_ is MR effective orifice area.

##### Pulmonary flow

The pulmonary valve flow waveform was modeled using a rectified sine curve with duration *t*_*ee*_ and amplitude Q_*MPV*_ as follows:

(9) QPV(t)=QMPVsin(πttee),t≤t;eeQPV(t)=0,tee<t≤T

where, Q_*MPV*_, t_*ee*_ and T are mean flow rate of the pulmonary valve, end-ejection time and cardiac cycle duration, respectively. Forward left ventricular outflow tract stroke volume (*Forward LVOT-SV*) was the sole input flow condition in this study. Indeed, the mean flow rate of the pulmonary valve (Q_*MPV*_) was optimized so that the lump-parameter algorithm replicates the measured *Forward LVOT*-SV.

#### Input Parameters and Patient-Specific Parameter Estimation

Both C3VI-CT and C3VI-DE algorithms use the following input parameters: forward left ventricular outflow tract stroke volume (*Forward LVOT-SV*), cardiac cycle duration (T), ejection time (T_*EJ*_), ascending aorta area (*A*_*AO*_), left ventricle outflow tract area (*A*_*LVOT*_), aortic valve effective orifice area (*EOA*|_*AV*_), mitral valve effective orifice area (*EOA*|_*MV*_), grading of the severity of aortic, and mitral valves regurgitation and systolic and diastolic blood pressures.

##### Flow inputs

Both C3VI-CT and C3VI-DE use only one measured flow parameter as an input: forward left ventricle stroke volume (*Forward LVOT-SV*). *Forward LVOT-SV* is defined as the volume of blood that passes through the LVOT cross sectional area every time the heart beats.

##### C3VI-CT

Forward LVOT-SV measured using CT is defined (Equation 10) as follows:

(10) ForwardLVOT-SV=EDV-ESV

where, *EDV* and *ESV* are the end diastolic volume and the end systolic volume, respectively. Using CT data, we have estimated end diastole phase and end systole phase by tracking the images and the spatial position of the mitral valve and aortic valve leaflets as well as the left ventricle. Therefore, the very first image after aortic-valve closure was deemed as the end systole (beginning of diastole) and the very first image after mitral-valve closure was considered as the end diastole (beginning of systole). We segmented and reconstructed the 3-D geometries of the complete ventricle in patients in both pre and post-TAVR using CT images and ITK-SNAP (version 3.8.0-BETA) (Yushkevich et al., 2006), a 3-D image processing and model generation software package (Figure 1C). We then, using an in house Matlab code, calculated the left ventricle volume at the end systole and end diastole after reconstructing the 3-D shape using CT data. We used smoothing procedure for the surfaces. The smoothing procedure mainly removed the effect of trabeculae and papillary muscles, which has been shown to have negligible influence on the ventricle hemodynamics (Vedula et al., 2016). Change in the volume due to smoothing was less than 3% in all patients.

##### C3VI-DE

*Forward LV-SV* measured using DE is defined as the following (Keshavarz-Motamed, 2020):

(11) $$ForwardLV-SV=A_{LVOT}VTI_{LVOT}=\frac{\pi {\left(D_{LVOT}\right)}^{2}}{4}VTI_{LVOT}$$

where, *D*_*LVOT*_, *A*_*LVOT*_, and *VTI*_*LVOT*_ are LVOT diameter, LVOT area, and LVOT velocity-time integral, respectively.

##### Time inputs

Cardiac cycle time (T) and ejection time (T_*EJ*_) were measured using Doppler echocardiography and ECG-Gated CT to be used in C3VI-DE and C3VI-CT, respectively.

##### Aortic valve and mitral valve inputs

To model blood flow in forward direction, both C3VI-CT and C3VI-DE require aortic valve effective orifice area (*EOA*|_*AV*_), mitral valve effective orifice area (*EOA*|_*MV*_), ascending aorta area (*A*_*AO*_) and left ventricle outflow tract area (*A*_*LVOT*_).

##### C3VI-CT

We segmented and reconstructed the 3-D geometries of the aortic and mitral valves, ascending aorta and LVOT section in C3VI patients in both pre and post-TAVR using CT images and ITK-SNAP (version 3.8.0-BETA) (Yushkevich et al., 2006) (Figure 1C). We calculated *EOA*|_*AV*_, *EOA*|_*MV*_, *A*_*AO*_ *and A*_*LVOT*_ using an in house Matlab code, after reconstructing the 3-D shape using CT data.

##### C3VI-DE

*EOA*|_*AV*_, *A*_*AO*_, *A*_*LVOT*_ were calculated using the following equations (Keshavarz-Motamed, 2020):

(12) $${EOA|}_{AV}=\frac{ForwardLVOT-SV}{VTI_{AO}}$$

(13) $$A_{AO}=\frac{\pi {\left(D_{AO}\right)}^{2}}{4}$$

(14) $$A_{LVOT}=\frac{\pi {\left(D_{LVOT}\right)}^{2}}{4}$$

where, *VTI*_*AO*_, *D*_*AO*_, and *D*_*LVOT*_ are the velocity time integral in the ascending aorta (amount of the blood flow going through the aorta), ascending aorta diameter and LVOT diameter, respectively.

Moreover, mitral valve is approximately an ellipse and its area was quantified using the following equation where d_1_ and d_2_ are mitral-valve diameters measured in the apical two-chamber and apical four-chamber views, respectively (Keshavarz-Motamed, 2020).

(15) $${EOA|}_{MV}=\frac{\pi d_{1}d_{2}}{4}$$

##### Grading of aortic and mitral valve regurgitation severity inputs

To model blood flow in the reverse direction, both C3VI-CT and C3VI-DE require grading of aortic and mitral valve regurgitation severity (e.g., regurgitant effective orifice area of aortic valve and regurgitant effective orifice area of mitral valve) [(see Keshavarz-Motamed, 2020) for all details)]. C3VI-CT uses CT data for all the mentioned input parameters, however, it cannot provide measurements for grading of aortic and mitral valve regurgitation severity, which are measured using DE (Keshavarz-Motamed, 2020). We therefore use grading of aortic and mitral valve regurgitation severity measured by DE for both C3VI-CT and C3VI-DE.

##### Systolic and diastolic blood pressures

Systolic and diastolic blood pressures measured using a sphygmomanometer are additional input parameters for both C3VI-CT and C3VI-DE.

##### Parameter estimation for systemic circulation

Parameters R_*SA*_, C_*SAC*_, and C_*ao*_ were optimized so that the aorta pressure calculated using the model matched the patient’s systolic and diastolic brachial pressures measured using a sphygmomanometer (see section “Computational Algorithm” and section “Patient-Specific Response Optimization” for details) for both C3VI-CT and C3VI-DE.

##### Simulation execution

Please see the section “Computational Algorithm” for both C3VI-CT and C3VI-DE calculations.

#### Computational Algorithm

The lumped-parameter algorithm was analyzed numerically by creating and solving a system of ordinary differential equations in Matlab Simscape (MathWorks, Inc.), supplemented by additional functions written in Matlab and Simscape. Matlab’s ode23t trapezoidal rule variable-step solver was used to solve the system of differential equations with an initial time step of 0.1 milliseconds. The convergence residual criterion was set to 10^–6^. Initial voltages and currents of capacitors and inductors were set to zero. The model was run for several cycles (around 50 cycles) to reach steady state before starting the response optimization process described below. In order to generate a signal to model LV elastance, a double Hill function representation of a normalized elastance curve for human adults was used (Mynard et al., 2012; Broomé et al., 2013). This elastance formulation was shown to completely represent the LV function independent of its pathological condition. Simulations started at the onset of isovolumic contraction. The instantaneous LV volume, V(t), was calculated using the time varying elastance (Equation 1) and LV pressure, P_*LV*_. Subsequently, the LV flow rate was calculated as the time derivative of the instantaneous LV volume. The same method was used to obtain the left-atrium volume, pressure and flow rate. P_*LV*_ was initially calculated using the initial values of the model input parameters from Table 1. The *Forward LVOT-SV* was calculated using the lumped-parameter model and then fitted to the one measured (Equation 10) by optimizing Q_*MPV*_ (as detailed below). Finally, for each patient R_*SA*_, C_*SAC*_, and C_*ao*_ were optimized to fit the aortic pressure from the model to the patient systolic and diastolic pressures measured using a sphygmomanometer.

#### Patient-Specific Response Optimization

The parameters of the lumped parameter algorithm are listed in Table 1. Some of the parameters were considered constant based on the previous studies in the literature or based on the rationale given below and their values are reported in Table 1. Additionally, the parameters that were measured in each patient are indicated in that table. To precisely replicate the body conditions of individual patients, as described below, four parameters of the lumped parameter algorithm were optimized so that the model replicated the physiological measurements performed in the patient. Simulink Design Optimization toolbox was used to optimize the response of the lumped-parameter model using the trust region reflective algorithm implemented in Matlab fmincon function. The response optimization was performed in two consecutive steps with tolerances of 10^–6^ (Figure 2, flow chart).

The mean flow rate of the pulmonary valve, Q_*MPV*_, could not be measured or computed using CT and cannot be reliably measured using Doppler echocardiography. However, because *Forward LVOT-SV* can be measured reliably using Doppler echocardiography and can be computed using CT, in the first step of optimization, Q_*MPV*_ was optimized to minimize the error between the *Forward LVOT-SV* calculated by the lumped-parameter algorithm and the one measured in each patient reliable using DE.

In the second step, R_*SA*_, C_*SAC*_, and C_*ao*_ were optimized so that maximum and minimum of the aorta pressures were equal to the systolic pressure and diastolic pressure, respectively, measured using a sphygmomanometer in each patient. Because the left ventricle confronts the total systemic resistance and not the specific resistances, and the systemic arteries resistance (*R*_*SA*_) is one order of magnitude greater than both the aortic resistance (*R*_*ao*_) and systemic vein resistance (*R*_*SV*_), we considered *R*_*ao*_ and *R*_*SV*_ as constants and optimized *R*_*SA*_ as the main contributor of the total systemic resistance (Keshavarz-Motamed et al., 2011, 2012, 2014, 2015; Benevento et al., 2015; Keshavarz-Motamed, 2020; Sadeghi et al., 2020). C_*ao*_ was considered to be 0.6 of C_*SAC*_ because 60% of the total arterial compliance lives in the proximal aorta (Stergiopulos et al., 1999).

In addition, we performed a comprehensive parameter sensitivity analysis that discovered negligible effects of changes in the pulmonary parameters (e.g., C_*PVC*_) on the lumped parameter model output variables (Keshavarz-Motamed, 2020). Therefore, we did not include these pulmonary parameters in the parameter-optimization process and counted them as constants (Table 1).

### Study Population

Forty-nine deidentified and anonymous C3VI patients with severe aortic valve stenosis who underwent TAVR (see Table 2 for patients characteristics) between 2011 and 2018 at St. Joseph’s Healthcare and Hamilton General Hospital (Hamilton, ON, Canada) and Hospital Universitario Marques de Valdecilla (IDIVAL, Santander, Spain) were considered (Keshavarz-Motamed, 2020; Keshavarz-Motamed et al., 2020). The selections were done by operators blinded to the objectives and contents of this study. Informed consent was obtained from all participants. The protocols were reviewed and approved by the Institutional Review Boards of each institution as follows: the Hamilton Integrated Research Ethics Board (HiREB) of Hamilton Health Sciences and St. Joseph’s Healthcare, both affiliated to McMaster University and Comité de ética de la investigación con medicamentos de Cantabria of the Hospital Universitario Marques de Valdecilla. All methods and measurements were conducted in accordance with pertinent guidelines and regulations, e.g., guidelines of the American College of Cardiology and American Heart Association. Cardiac catheterizations were performed only in pre intervention status. The patient medical records were used to collect demographic and procedural data (see Table 1 for details). Data was acquired at two time points: pre-procedure and 90-days post-procedure.

**TABLE 2:** Patient characteristics.

		**Pre intervention Mean ± SD (*n* = 49)**	**Post intervention Mean ± SD (*n* = 49)**
**Ventricular indices – DE findings**			
	Ejection fraction, %	53.5 ± 12.7	61 ± 14.6
	Heart rate, bpm	70.7 ± 9.5	68 ± 11.8
	Stroke volume, mL	48.3 ± 11.7	44.5 ± 15.5
	NYHA classifications ≥ grade 2	82%	76%
**Valvular indices – DE findings**			
	Aortic valve effective orifice area (cm^2^)	0.58 ± 0.16	1.75 ± 0.4
	Mean aortic valve gradient, mmHg	51.52 ± 13.6	11.1 ± 6.1
	Maximum aortic valve gradient, mmHg	84.5 ± 21.32	20.4 ± 10.28
	Aortic valve disease type	Tricuspid: 46; Bicuspid: 3	None
	Aortic valve regurgitation ≥ grade 2	48%	5%
	Mitral valve regurgitation ≥ grade 2	19%	20%
**Vascular indices – Sphygmomanometer**			
	Brachial systolic blood pressure, mmHg	139 ± 22.5	135 ± 16.8
	Brachial diastolic blood pressure, mmHg	79 ± 11.7	68 ± 10.3
**Patient description**			
	Mean age, years; Gender	64.5 ± 5.5; (Female: 36%)	Same as pre TAVR
	Mean weight, kg; Mean height, cm	73.4 ± 12.8; 165.7 ± 9.6	71.6 ± 10.5; 165.7 ± 9.6
	Body surface area, m^2^	1.73 ± 0.14	Not available
	Body mass index, kg/m^2^	31.9 ± 21.5	Not available
	EuroScore II	7.2 ± 5.33	Not available
	STS mortality rate	6.89 ± 4.45	Not available
**Associated cardiovascular lesions**			
	Previous percutaneous coronary intervention	39%	Same as pre TAVR
	Previous coronary artery bypass grafting	30%	Same as pre TAVR
	Previous myocardial infarction	19%	Same as pre TAVR
	Previous stroke	1%	Same as pre TAVR
	Atrial fibrillation	26%	Same as pre TAVR
	Cerebrovascular accident	5%	Same as pre TAVR
	Peripheral vascular disease	38%	Same as pre TAVR
	Hypertension	82%	78%

*Changes in hemodynamic metrics from baseline to post-intervention.*

### Statistical Analysis

All results were expressed as mean ± standard deviations (SD). Statistical analyses were performed using SigmaStat software (Version 3.1, Systat Software, San Jose, CA, United States). Coefficient of determination, *R*^2^, was used to quantify the quality of linear regressions. Statistically significant differences between two datasets were assessed using two-sample *t*-test at 1% significance level.

## Results

### C3VI-DE and C3VI-CT vs. Clinical Cardiac Catheterization Data

**FIGURE 3:**
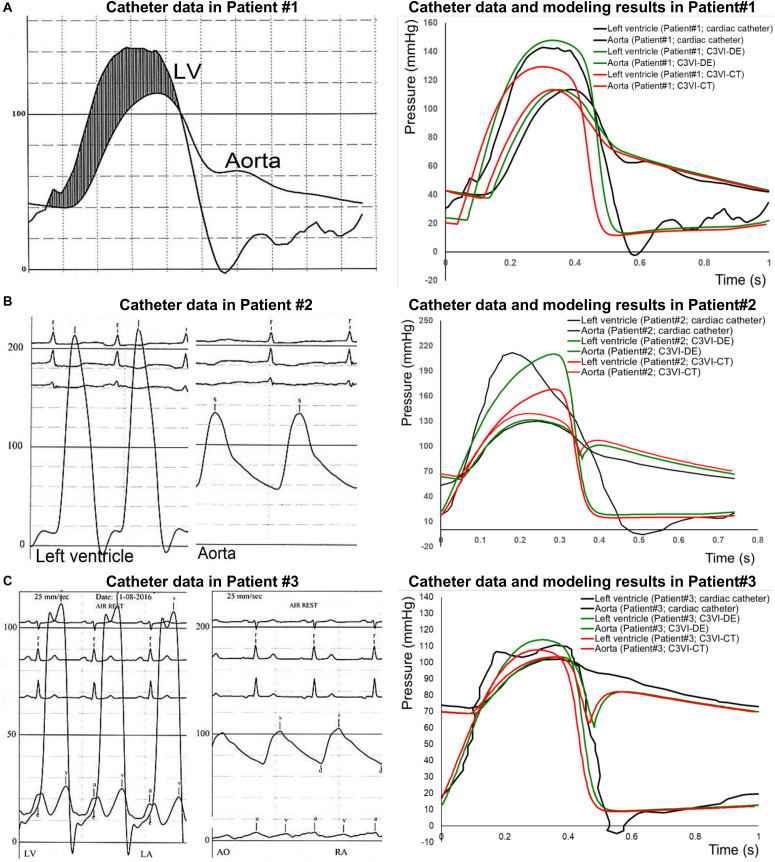
Pressure waveform comparison (C3VI-CT & C3VI-DE *vs.* cardiac catheter): The instantaneous C3VI-DE pressure compared favorably with cardiac catheter pressure in all subjects. Conversely, results from C3VI-CT do not precisely agree with catheter measurements. **(A)** Patient #1; **(B)** Patient #2; **(C)** Patient #3.

**FIGURE 4:**
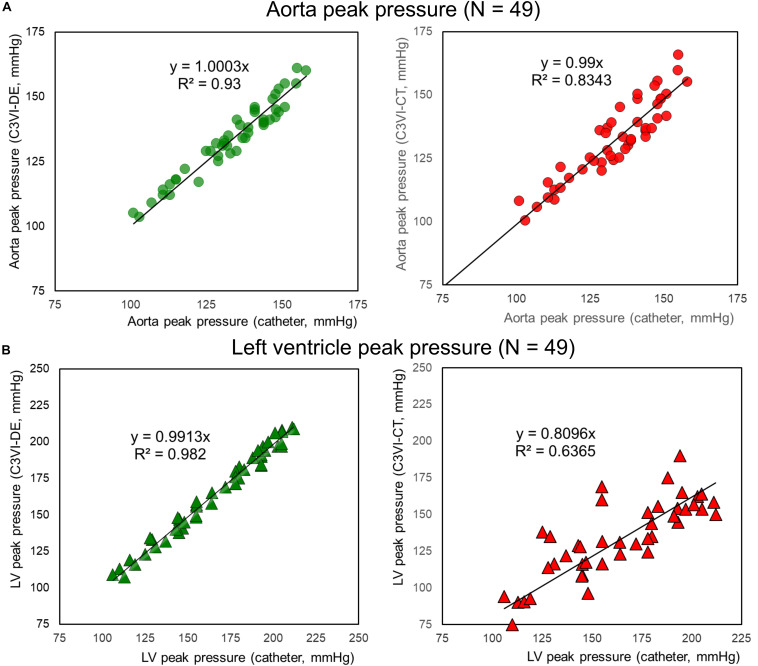
Peak pressure correlation (C3VI-CT & C3VI-DE *vs.* cardiac catheter). Peak pressures calculated by C3VI-DE correlated well with catheter measurements in all forty-nine C3VI patients, described by high coefficients of determination. In contrast with C3VI-DE, peak pressures obtained from C3VI-CT are incompatible with the catheter measurements, described by low coefficients of determination. **(A)** Aorta. **(B)** Left ventricle.

Validation with clinical cardiac catheterization is a gold standard and the highest-level validation that is possible in patients in the field of cardiovascular mechanics. However, because of its invasive nature, catheter data are rare and collecting a useable dataset are incredibly rare. It is important to note that from a fluid mechanics point of view, in incompressible flow the relationship between pressure and velocity is well defined and therefore from catheter pressure data, the velocity can be easily obtained. In the complex time-varying cardiovascular system, in which many phenomena interact with one another, having a model that replicates the catheter data in each patient, shows the validity of the model to the highest degree. Our results show that C3VI-DE can non-invasively quantify pulsatile flow and pressure throughout the heart in C3VI patients and provide instantaneous quantities such as left ventricle and aorta pressures. Conversely, C3VI-CT cannot accurately obtain these quantities (Figures 3, 4).

#### Pressure Waveforms

The instantaneous pressure computed by C3VI-DE were compared with clinical cardiac catheter pressure measurements in all forty-nine C3VI patients. Figure 3 shows the comparison of C3VI-DE calculations with catheter data in 3 of the 49 patients (Patients #1, #2, and #3). C3VI-DE results are in qualitative agreement with catheter measurements, e.g., similar waveform shape as well as specific wave elements such as the amplitude and timing of the systolic peak. Quantitatively, results computed by C3VI-DE had an average RMS error of 11.8 mmHg and 9.9 mmHg in the LV and aorta pressures, respectively (*n* = 49). Conversely, results from C3VI-CT do not precisely agree with catheter measurements with the average RMS errors of 64.5 mmHg and 12.7 mmHg in the left ventricle and aorta pressures, respectively (*n* = 49).

#### Peak Pressure

The peak pressures obtained from C3VI-DE (LV: 164.5 ± 30.7 mmHg; aorta: 133.88 ± 14.25 mmHg) are in close agreement with catheter measurements (LV: 165.9 ± 30.9 mmHg, aorta: 133.75 ± 14.67 mmHg) in all forty-nice C3VI patients (Figure 4). The high coefficients of determination (LV: *R*^2^ = 0.982; aorta: *R*^2^ = 0.933; Figure 4) indicate a strong correlation between C3VI-DE and cardiac catheter measurements, with maximum relative errors of 4.49% for aorta pressure and 4.33% for LV pressure in all forty-nine patients. In contrast with those obtained from C3VI-DE, peak pressures obtained from C3VI-CT (LV: 143.4 ± 27.5 mmHg, aorta: 134.4 ± 14.5 mmHg) are incompatible with the catheter measurements. Its low coefficients of determination (LV: *R*^2^ = 0.63; aorta: *R*^2^ = 0.83; Figure 4) indicate a weak correlation between C3VI-CT and catheter measurements, with maximum relative errors of 31.4% and 7.8% for LV and aortic pressures, respectively, in all C3VI subjects.

### C3VI-DE vs. C3VI-CT: Input Parameters to the Model

**FIGURE 5:**
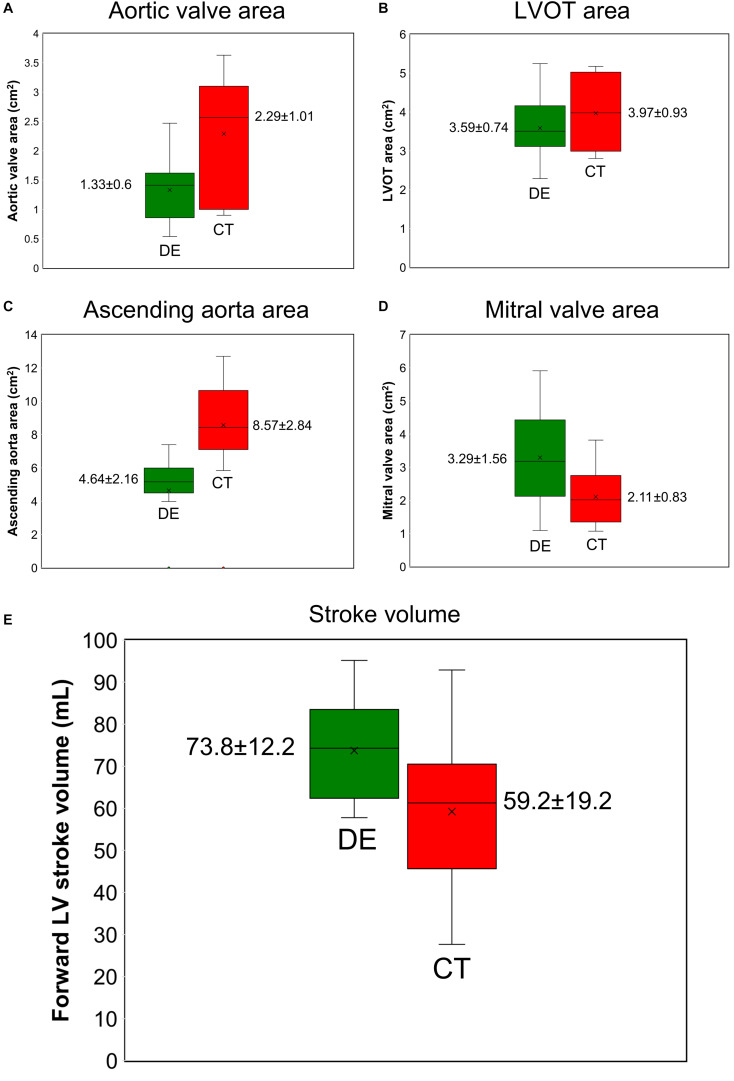
Doppler echocardiography measurements *vs.* computed tomography measurements (*N* = 49). **(A)** aortic valve effective orifice area; **(B)** LVOT area; **(C)** ascending aorta area; **(D)** mitral valve effective orifice area; **(E)** forward LVOT stroke volume. DE and CT generated significantly different results for aortic valve effective orifice area, LVOT area, ascending aorta area, mitral valve effective orifice area, and forward LVOT stroke volume as the two sample *t*-test rejected the null hypothesis at 0.01 significance level.

The developed algorithm uses the following input parameters: forward LVOT stroke volume, cardiac cycle duration, ascending aorta area, LVOT area, aortic valve effective orifice area, mitral valve effective orifice area, and grading of aortic and mitral valves regurgitation severity. While for C3VI-DE all of these parameters are reliably measured using DE, for C3VI-CT they are measured using CT, except for grading of aortic and mitral valve regurgitation severity, which are measured using DE since CT cannot provide these measurements. The other input parameters of the model are systolic and diastolic blood pressures, which are measured using a sphygmomanometer for both C3VI-DE and C3VI-CT. Figure 5 shows that when the measurements of the input parameters were performed using CT data, aortic valve effective orifice area, LVOT area, and ascending aorta area were significantly higher than those measured using DE, while the forward LVOT stroke volume and mitral valve effective area were lower than the ones measured using DE. Table 3 shows the maximum variations of the computed LV workload and LV peak pressure, averaged over all patients, obtained from one-parameter-at-a-time sensitivity analysis of ±30% relative to the baseline. As shown in Table 3, the LV worklaod and LV peak pressure are greatly sensitive to the forward LVOT stroke volume, among all of the input parameters of the model, and consequently the underestimated forward LVOT stroke volume obtained from the CT data can introduce an error in the calculated LV workload.

**TABLE 3:** Maximum variation of the computed LV workload and LV peak pressure.

**Input parameters of the algorithm measured using CT or DE**	**Abbreviation**	**Maximum variations of computed LV workload and LV peak pressure (*N* = 49)**
Forward left ventricular outflow tract stroke volume	Forward LVOT-SV	58% and 51%
Cardiac cycle duration	T	14.5% and 11%
Ascending aorta area	A_*AO*_	0.68% and 0.5%
LVOT area	A_*LVOT*_	0.7% and 0.65%
Aortic valve effective orifice area	EOA_*AV*_	14% and 18%
Mitral valve effective orifice area	EOA_*MV*_	1.4% and 0.9%
**Input parameters of the algorithm measured using DE**		
Regurgitant effective orifice area of the aortic valve	EOA_*AR*_	19% and 10.6%
Regurgitant effective orifice area of the mitral valve	EOA_*MR*_	11.5% and 4.5%
**Input parameters of the algorithm measured using sphygmomanometers**		
Systolic pressure	P_*SYS*_	1.2% and 0.85%
Diastolic pressure	P_*DIAS*_	1% and 0.9%

*Results were obtained from one-parameter-at-a-time sensitivity analysis (±30%) relative to the baseline, averaged over all patients (*N* = 49). LV workload, the integral of LV pressure and its volume change, is greatly sensitive to the forward LVOT stroke volume, among all of the input parameters of the algorithm.*

### C3VI-DE vs. C3VI-CT: Model Outputs (Hemodynamics Metrics of Circulatory and Cardiac Function)

**FIGURE 6:**
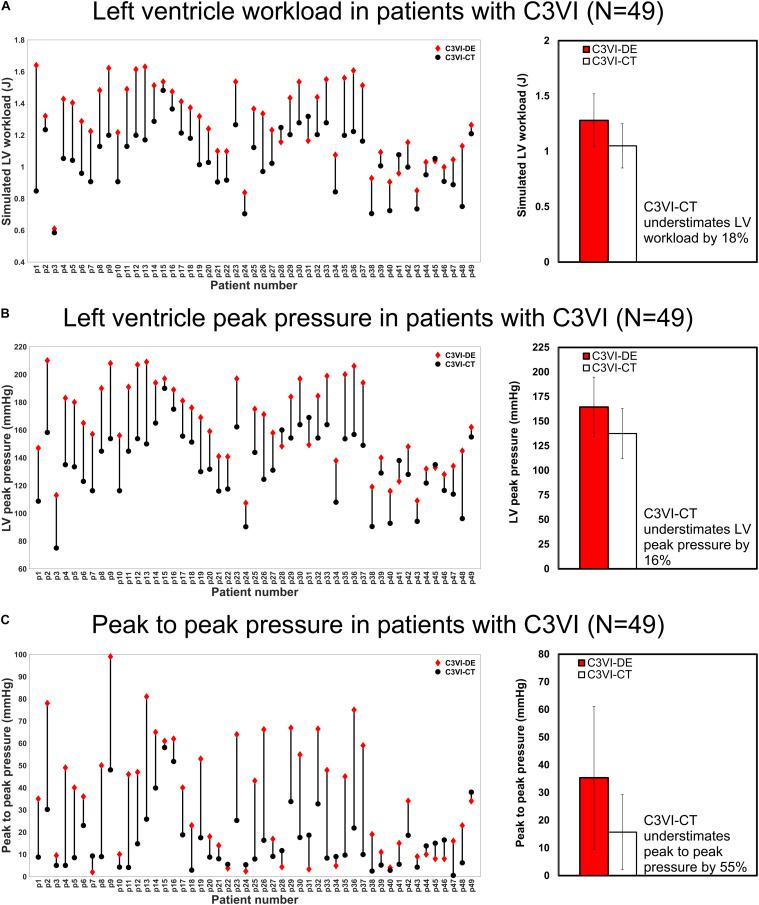
Changes in hemodynamics assessments calculated by C3VI-DE and C3VI-CT in patients with C3VI (*N* = 49). **(A)** LV workload; **(B)** LV peak pressure; **(C)** Peak to peak pressure. Two-sample *t*-test showed that calculations of C3VI-DE and C3VI-CT for all three variables (LV workload, LV peak pressure and peak to peak pressure) are significantly different at 0.01 significance level.

**FIGURE 7:**
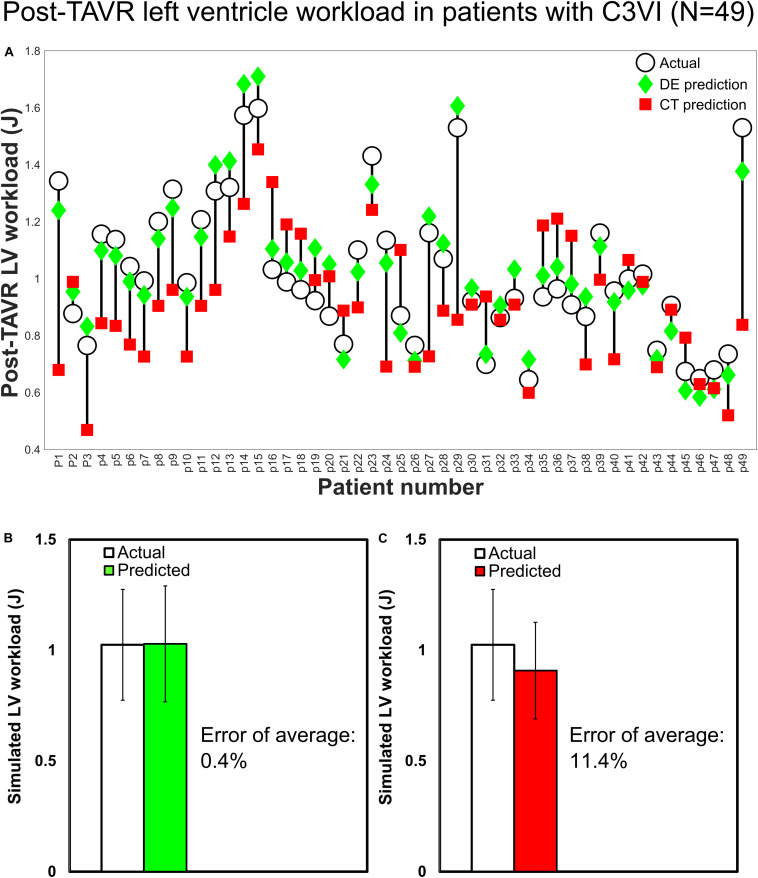
Changes in predicted LV workload after intervention and actual post-intervention LV workload in patients with C3VI (*N* = 49). **(A)** Computed by C3VI-DE and C3VI-CT. **(B)** Computed by C3VI-DE. **(C)** Computed by C3VI-CT. The two-sample *t*-test rejects the null hypothesis that the prediction and the actual values of the post-intervention LV workload have equal means for C3VI-CT predictions but not for C3VI-DE predictions at 0.01 significance.

Figure 6 shows that the calculated hemodynamics metrics of circulatory and cardiac function (e.g., LV workload, LV peak pressure and peak to peak pressure gradient) were substantially different when the measurements of the input parameters were performed using DE rather than CT. Compared to C3VI-DE, C3VI-CT underestimates the LV workload, LV peak pressure and peak to peak pressure gradient (the difference between LV peak pressure and aorta peak pressure) by 18%, 16%, and 55%, respectively (average in *N* = 49). Moreover, we used the pre-intervention states (both from DE and CT) of the patients, virtually performed intervention in the models and used our framework to predict the patient state post intervention. Figure 7 compares the actual post-intervention LV workload with the LV workload that our framework predicted that all patients would have after the intervention (patients with C3VI underwent TAVR; *N* = 49). We observed quantitative agreement, resulted from, between the post-intervention LV workload predicted using C3VI-DE and the actual post-intervention LV workload in all C3VI subjects (error of average: 0.4%, *N* = 49; Figure 7) which demonstrates the validity of the C3VI-DE model and its predictive capability. However, there is no firm quantitative agreement between the post-intervention LV workload predicted using C3VI-CT and the actual post-intervention LV workload in C3VI patients (error of average: 11.4%, *N* = 49; Figure 7).

## Discussion

Because of high individual differences in the anatomy and pathophysiology of patients, planning individualized treatment requires patient-specific diagnosis. Hemodynamics quantification in C3VI plays an essential role in precise and early diagnosis (Marsden, 2013; Khalafvand et al., 2014; Di Carli et al., 2016), however, present diagnostic methods are limited and cannot quantify hemodynamics of C3VI (Marsden, 2013; Di Carli et al., 2016; Anvari et al., 2021).

As the need for patient-specific diagnostic methods continues to be studied, understanding the strengths and limitations of imaging modalities is crucial toward creating accurate diagnostic tools. Over the past decade, the use of medical imaging has exponentially increased (Smith-Bindman et al., 2012; Blecker et al., 2013), likely due to its technological advancements which is evident through the miniaturization of imaging devices and the dramatic increase in sensitivity and spatial resolution (Fleischmann et al., 2008; Smith-Bindman et al., 2012; Di Carli et al., 2016). In spite of astonishing advancements in medical imaging, medical imaging on its own cannot quantify local and global hemodynamics: (1) Phase-contrast magnetic resonance imaging (MRI): MRI can provide 3-D velocity and volumetric data throughout the cardiac cycle, making it a great tool for characterizing flow throughout the volume with a relatively high spatial resolution (Fleischmann et al., 2008; Shen et al., 2021). On the downside, MRI has a lower temporal resolution (20 ms highest) than Doppler echocardiography does, and is the most costly of the compared imaging modalities (Picano, 2005; Watson et al., 2018). In addition, Gadolinium contrast agent, used in approximately 1 in 3 MRI scans to increase the image clarity, is toxic and may lead to the development of Nephrogenic Systemic Fibrosis in patients with severe renal failure (Kuo et al., 2007; Paterson et al., 2013). However, 4D flow MRI is an emerging technology to allow comprehensive assessment of cardiac function, vascular and valvular function (Zhong et al., 2019). Most importantly, although use MRI is limited in patients with implanted medical devices as they remain a major risk during the examination (Orwat et al., 2014), some devices [e.g., MRI-conditional pacemakers; Saunderson et al. (2020) and (Saunderson et al., 2020)] may be used in MRI environment if certain conditions are fulfilled. However, the possibility, safety and reliability of 4-D flow MRI remain to be confirmed in patients with implanted cardiac devices. As Saunderson et al. (Saunderson et al., 2020) mentioned, larger studies are required to fully evaluate safety of 4D flow MRI across a wider range of cardiovascular implanted devices; (2) Computed tomography (CT): CT scans allows for 3D and 4D visualization and measurement of complex anatomy as well as flexible structures at high spatial resolution (Villarraga-Gómez et al., 2018). Dual source CT has poor temporal resolution with the highest resolution outputs of 83 ms, which is the lowest of the compared modalities, thus requiring slow and steady heart rates to yield a clear image (Lin and Alessio, 2009; Sabarudin and Sun, 2013; Watson et al., 2018). Additionally, due to the ionizing radiation, receiving multiple scans increases the risk of developing cancer by 2.7–12% for the general population and up to triple the risk of brain tumors and leukemia for pediatric patients. Furthermore, CT typically requires the use of an iodine-based contrast agent which, in rare cases, may induce anaphylaxis or contrast-induced nephropathy (Andreucci et al., 2014; Faggioni and Mehran, 2016; Rehman and Makaryus, 2019). More importantly, CT cannot measure any (local and global) hemodynamic parameters; (3) Doppler echocardiography (DE): DE provides functional, real-time information regarding cardiac geometry, instantaneous flow and pressure gradients (Anavekar and Oh, 2009; Steeds, 2011). DE can detect structural abnormalities as well as assess contractility and ejection fraction, at an excellent temporal resolution of < 4 ms and has an infinitesimal risk-to-benefit ratio (Papolos et al., 2016). As a result, DE remains the gold standard for assessing cardiac function, and is essential for basic and clinical cardiovascular research (Anavekar and Oh, 2009; Steeds, 2011; Parra and Vera, 2012). Moreover, DE is the least costly of the compared imaging modalities as well as the most widely available. Despite its versatility and potential, DE cannot precisely evaluate local and global hemodynamics, or provide breakdown contributions of each component in cardiovascular disease (Anavekar and Oh, 2009; Scantlebury Dawn et al., 2013). Such information has a high clinical importance for planning advanced treatments for C3VI patients.

We recently developed a non-invasive, image-based, patient-specific diagnostic and monitoring lumped parameter modeling framework for C3VI patients (called C3VI-DE) which uses limited input parameters, measured using DE reliably and a sphygmomanometer (Keshavarz-Motamed, 2020). Additionally, in this study, we have developed another computational framework that used the same lumped-parameter model core in conjunction with input parameters measured using CT and a sphygmomanometer (called C3VI-CT). *In this study, we focused on comparing data generated from DE and CT as they are commonly used in clinics for patients with C3VI and severe aortic stenosis who received TAVR.* In this paper, we compared accuracy of the results obtained using C3VI-DE and C3VI-CT against catheterization data in forty-nine C3VI patients with severe aortic stenosis who underwent TAVR with substantial inter- and intra-patient variability covering a wide range of diseases to determine with which modality the framework can yield the most accurate results. Based on our analysis, we found that results from C3VI-DE are in qualitative and quantitative agreement with catheter measurements, whereas results from C3VI-CT do not agree with catheter measurements.

Although DE suffers from operator dependence and is only able to provide a single component of the flow velocity, the input parameters that are used in C3VI-DE (detailed in section “Materials and Methods”) can be reliably measured using DE. Furthermore, our proposed method decreases the operator dependence of DE by providing many quantitative measures that are obtained independent from the operator and are not accessible otherwise by any other method or imaging modality. CT has a high spatial resolution and can provide anatomical information with a high accuracy (Villarraga-Gómez et al., 2018), however, it has a low temporal resolution (Maleki and Esmaeilzadeh, 2012; Watson et al., 2018; Rehman and Makaryus, 2019). All input parameters obtained from CT images to be used in C3VI-CT (detailed in section “Materials and Methods”) were measured at the closest instance to end systole and end diastole but because of poor time resolution, the situation of the left ventricle and valves are not known at the exact end systole and end diastole instances. we have estimated end diastole phase and end systole phase by tracking the images and the spatial position of the mitral valve and aortic valve leaflets as well as the left ventricle. Therefore, the very first image after aortic-valve closure was deemed as the end systole (beginning of diastole) and the very first image after mitral-valve closure was considered as the end diastole (beginning of systole). Although DE images do not have as high spatial resolution as CT images, the input parameters that are required for C3VI-DE are among the quantities that can be reliably measured in DE images. Furthermore because of high temporal resolution of DE, the end diastole and end systole instances can be accurately determined. We believe that reliably measured input parameters at a high temporal resolution using DE enabled more accurate results in C3VI-DE than the ones obtained from C3VI-CT based on input parameters that were affected by the poor temporal resolution of CT. Please note in this study, all measurements of the input parameters for both C3VI-DE and C3VI-CT were verified by two cardiologists.

The C3VI-DE framework is an innovative non-invasive diagnostic and monitoring tool that can investigate and quantify effects of C3VI components on cardiac function and the circulatory system. C3VI-DE is centered around calculations of local hemodynamics (fluid dynamics of the circulatory system) and global hemodynamics (cardiac function and hemodynamics). Furthermore, by decomposing the global hemodynamics into the individual contributions of each C3VI disease constituent, it can help predicting the effects of interventions as well as planning for the suitable sequence of interventions and for making critical clinical decisions with life-threatening risks. C3VI-DE is capable of monitoring both the cardiac and vascular conditions and can be used for their diagnosis with direct clinical relevance.

## Limitations

This study was performed using data collected in 49 patients with C3VI. Future studies must confirm the conclusion of this study about C3VI-DE and C3VI-CT in a larger population of C3VI patients. In addition, future studies should further investigate the C3VI-CT algorithm with a higher temporal resolution of CT data (if becomes available).

## Data Availability Statement

The raw data supporting the conclusions of this article will be made available by the correspondence author upon request.

## Ethics Statement

The studies involving human participants were reviewed and approved by the Institutional Review Boards of each institution as follows: the Hamilton Integrated Research Ethics Board (HiREB) of Hamilton Health Sciences and St. Joseph’s Healthcare, both affiliated to McMaster University and Comité de ética de la investigación con medicamentos de Cantabria of the Hospital Universitario Marques de Valdecilla. The patients/participants provided their written informed consent to participate in this study.

## Author Contributions

MB and SB: data collection and analysis, interpretation of data, and manuscript writing. SK, JT, SV, ED, and MM: clinical data collection and analysis. ZK-M: conception and design, algorithm development (both C3VI-DE and C3VI-CT), data analysis, interpretation of data, manuscript writing, critical revision final approval of the manuscript, and supervise the research. All authors read and approved the final manuscript.

## Conflict of Interest

The authors declare that the research was conducted in the absence of any commercial or financial relationships that could be construed as a potential conflict of interest.
